# Supplementary material for: A UMLS-based spell checker for natural language processing in vaccine safety
Source: BMC Med Inform Decis Mak. 2007 Feb 12;7:3. doi: 10.1186/1472-6947-7-3 (PMC1805499; doi:10.1186/1472-6947-7-3)
Supplement: Additional file 13 — RAP application directory. Sets up directory for RAP (RDF application for PHP) source code [file 1472-6947-7-3-S13.gz › rap/doc/phpdoc/__filesource/fsource_model__modelMemModel.php.html]

xml version="1.0" encoding="iso-8859-1"?


File Source for MemModel.php


# Source for file MemModel.php

Documentation is available at MemModel.php

```
1. <?php

3. // ----------------------------------------------------------------------------------
4. // Class: MemModel
5. // ----------------------------------------------------------------------------------


9. /**
10. * A MemModel is an RDF Model, which is stored in the main memory.
11. * This class provides methods for manipulating MemModels.
12. *
13. * <BR><BR>History:<UL>
14. * <li>
15. * <li>05-04-2005                : bugfixes in findFirstMatchingStatement() and findFirstMatchOff;
16. * <li>01-26-2005                : small bugfix in findFirstMatchingStatement();
17. * <LI>12-06-2004                 : improved namespace handling added (tobias.gauss@web.de)</LI>
18. * <LI>10-07-2004                 : Function findFirstMatchingStatement() added paramter $offset
19. *                                 to set an search offset
20. * <LI>09-09-2004                 : Function remove() bug removed: now removes ALL matching statements.
21. Function addWithoutDuplicates() now uses the contains() / add() functions.
22. Functions reify(),addModel(),unite(),subtract() changed to use the statement iterator.</LI>
23. * <LI>16-08-2004                 : Function getUniqueResourceURI() has been made faster</LI>
24. * <LI>08-06-2004                : Function findAsIterator() added</LI>
25. * <LI>06-13-2004                : Improved function index() which allows the definition of different search indexes</LI>
26. * <LI>03-29-2004                 : Function addModel() fixed, reindexing of triples in remove() added</LI>
27. * <LI>11-13-2003                 : Functions load, saveAs moved to class model
28. * <LI>11-12-2003                 : Function rdqlQueryAsIterator() added.</LI>
29. * <LI>07-31-2003                : Class renamed from Model to MemModel.
30. *                                 Variable BaseURI and Function getBaseURI() moved to Model.
31. *                                 Functions containsAll(), containsAny(), equals(), unite(),
32. *                                           subtract(), intersect(), addModel() can now
33. *                                           get DbModel as paramter.
34. *                                 Functions load() and saveAs() can now work with n3 format too.
35. *                                 Function rdqlQuery added. radol@gmx.de</LI>
36. * <LI>05-31-2003                : Functions load(), saveAs() and writeXX() findFirstMatchingStatement() added.
37. *                                    Changed function index() to a faster index type.</LI>
38. * <LI>05-28-2003                : Functions addWithoutDuplicates() and addModel() added, optimised bits and pieces.</LI>
39. * <LI>05-19-2003                : Function add() made more efficient, optimised bits and pieces. ggrimnes@csd.abdn.ac.uk</LI>
40. * <LI>05-19-2003                : Functions index() and isIndexed() added, ggrimnes@csd.abdn.ac.uk</LI>
41. * <LI>02-21-2003                : Function findCount() added.</LI>
42. * <LI>01-13-2003                : Function equals() made more efficient.</LI>
43. * <LI>09-10-2002                : First version of this class.</LI>
44. *
45. *
46. * @version  V0.9.1
47. * @author Chris Bizer <chris@bizer.de>
48. * @author Gunnar AAstrand Grimnes <ggrimnes@csd.abdn.ac.uk>
49. * @author Radoslaw Oldakowski <radol@gmx.de>
50. * @author Daniel Westphal <mail@d-westphal.de>
51. * @author Tobias Gau�    <tobias.gauss@web.de>
52. *
53. * @package model
54. * @todo nothing
55. * @access    public
56. */

58. class MemModel extends Model {

60. /**
61. * Triples of the MemModel
62. * @var        array
63. * @access    private
64. */
65. var $triples = array();

67. /**
68. * Array containing the search indices
69. * @var        array['INDEX_TYPE'][]['label'][]['PosInModel']
70. *
71. * @access   private
72. */
73. var $indexArr ;


76. /**
77. * depending on which index is used this variable is -1,0,1,2 or 3
78. *
79. * -1 : no index
80. *  0 : default indices over subject, predicate, object separate
81. *  1 : index over subject+predicate+object
82. *  2 : index over subject+predicate
83. *  3 : index over subject+object
84. *
85. * @var        int
86. * @access    private
87. */
88. var $indexed;


92. /**
93. * Array of namespaces
94. *
95. * @var     array
96. * @access    private
97. */
98. var $parsedNamespaces=array();


102. /**
103. * Constructor
104. * You can supply a base_uri
105. *
106. * @param string $baseURI
107. * @access    public
108. */
109. function MemModel($baseURI = NULL) {
110. $this->setBaseURI($baseURI);
111. $this->indexed = INDEX_TYPE;
112. }

114. /**
115. * Set a base URI for the MemModel.
116. * Affects creating of new resources and serialization syntax.
117. * If the URI doesn't end with # : or /, then a # is added to the URI.
118. * @param    string    $uri
119. * @access    public
120. */
121. function setBaseURI($uri) {

123. if ($uri != NULL) {
124. $c = substr($uri, strlen($uri)-1 ,1);
125. if (!($c=='#' || $c==':' || $c=='/' || $c=="\\"))
126. $uri .= '#';
127. }
128. $this->baseURI = $uri;
129. }


132. /**
133. * Number of triples in the MemModel
134. *
135. * @return    integer
136. * @access    public
137. */
138. function size() {
139. return count($this->triples);
140. }

142. /**
143. * Checks if MemModel is empty
144. *
145. * @return    boolean
146. * @access    public
147. */
148. function isEmpty() {
149. if (count($this->triples) == 0) {
150. return TRUE;
151. } else {
152. return FALSE;
153. };
154. }


157. /**
158. * Adds a new triple to the MemModel without checking if the statement is already in the MemModel.
159. * So if you want a duplicate free MemModel use the addWithoutDuplicates() function (which is slower then add())
160. *
161. * @param        object Statement    $statement
162. * @access    public
163. * @throws    PhpError
164. */
165. function add($statement) {
166. if (!is_a($statement, 'Statement')) {
167. $errmsg = RDFAPI_ERROR . '(class: MemModel; method: add): Statement expected.';
168. trigger_error($errmsg, E_USER_ERROR);
169. }

171. if($this->indexed != -1){
172. $this->triples[] = $statement;
173. end($this->triples);
174. $k=key($this->triples);
175. if($this->indexed==0){
176. // index over S
177. $this->_indexOpr($statement,$k,4,1);
178. // index over P
179. $this->_indexOpr($statement,$k,5,1);
180. // index over O
181. $this->_indexOpr($statement,$k,6,1);
182. }else{
183. $this->_indexOpr($statement,$k,$this->indexed,1);
184. }

186. }else{
187. $this->triples[] = $statement;
188. }
189. }


193. /**
194. * Checks if a new statement is already in the MemModel and adds the statement, if it is not in the MemModel.
195. * addWithoutDuplicates() is significantly slower then add().
196. * Retruns TRUE if the statement is added.
197. * FALSE otherwise.
198. *
199. * @param    object Statement    $statement
200. * @return    boolean
201. * @access    public
202. * @throws    PhpError
203. */
204. function addWithoutDuplicates($statement) {

206. if (!is_a($statement, 'Statement')) {
207. $errmsg = RDFAPI_ERROR . '(class: MemModel; method: addWithoutDuplicates): Statement expected.';
208. trigger_error($errmsg, E_USER_ERROR);
209. }

211. if (!$this->contains($statement)) {
212. $this->add($statement);
213. return true;
214. }else{
215. return false;
216. }
217. }

219. /**
220. * Removes the triple from the MemModel.
221. * TRUE if the triple is removed.
222. * FALSE otherwise.
223. *
224. * @param        object Statement    $statement
225. * @return    boolean
226. * @access    public
227. * @throws    PhpError
228. */
229. function remove($statement) {

231. if (!is_a($statement, 'Statement')) {
232. $errmsg = RDFAPI_ERROR . '(class: MemModel; method: remove): Statement expected.';
233. trigger_error($errmsg, E_USER_ERROR);
234. }
235. if($this->indexed==-1){
236. $pass=false;
237. foreach($this->triples as $key => $value) {
238. if ($this->matchStatement($value, $statement->subject(), $statement->predicate(), $statement->object())) {
239. unset($this->triples[$key]);
240. $pass= true;
241. }
242. }
243. return $pass;
244. }else{
245. $k= null;
246. if($this->indexed==0){
247. $pass=false;
248. $del=false;
249. while($del!=-1){
250. // index over S
251. $del=$this->_indexOpr($statement,$k,4,0);
252. // index over P
253. $this->_indexOpr($statement,$k,5,0);
254. // index over O
255. $this->_indexOpr($statement,$k,6,0);
256. if($del!=-1){
257. unset($this->triples[$del]);
258. $pass=true;
259. }
260. }
261. return $pass;
262. }else{
263. $pass=false;
264. $del=false;
265. while($del!=-1){
266. $del=$this->_indexOpr($statement,$k,$this->indexed,0);
267. if($del!=-1){
268. unset($this->triples[$del]);
269. $pass=true;
270. }
271. }
272. return $pass;
273. }
274. }
275. }

277. /**
278. * Short Dump of the MemModel.
279. *
280. * @access    public
281. * @return    string
282. */
283. function toString() {
284. return 'MemModel[baseURI=' . $this->getBaseURI() . ';  size=' . $this->size() . ']';
285. }

287. /**
288. * Dumps of the MemModel including all triples.
289. *
290. * @access    public
291. * @return    string
292. */
293. function toStringIncludingTriples() {
294. $dump = $this->toString() . chr(13);
295. foreach($this->triples as $value) {
296. $dump .= $value->toString() . chr(13);
297. }
298. return $dump;
299. }


304. /**
305. * Writes the RDF serialization of the MemModel as HTML.
306. *
307. * @access    public
308. */
309. function writeAsHtml() {
310. require_once(RDFAPI_INCLUDE_DIR.PACKAGE_SYNTAX_RDF);
311. $ser = new RdfSerializer();
312. $rdf =& $ser->serialize($this);
313. $rdf = htmlspecialchars($rdf, ENT_QUOTES);
314. $rdf = str_replace(' ', '&nbsp;', $rdf);
315. $rdf = nl2br($rdf);
316. echo $rdf;
317. }

319. /**
320. * Writes the RDF serialization of the MemModel as HTML table.
321. *
322. * @access    public
323. */
324. function writeAsHtmlTable() {
325. // Import Package Utility
326. include_once(RDFAPI_INCLUDE_DIR.PACKAGE_UTILITY);
327. RDFUtil::writeHTMLTable($this);
328. }


331. /**
332. * Writes the RDF serialization of the MemModel as HTML table.
333. *
334. * @access    public
335. * @return    string
336. */
337. function writeRdfToString() {
338. // Import Package Syntax
339. include_once(RDFAPI_INCLUDE_DIR.PACKAGE_SYNTAX_RDF);
340. $ser = new RdfSerializer();
341. $rdf =& $ser->serialize($this);
342. return $rdf;
343. }


346. /**
347. * Saves the RDF,N3 or N-Triple serialization of the MemModel to a file.
348. * You can decide to which format the model should be serialized by using a
349. * corresponding suffix-string as $type parameter. If no $type parameter
350. * is placed this method will serialize the model to XML/RDF format.
351. * Returns FALSE if the MemModel couldn't be saved to the file.
352. *
353. * @access    public
354. * @param     string     $filename
355. * @param     string     $type
356. * @throw   PhpError
357. * @return    boolean
358. */
359. function saveAs($filename, $type ='rdf') {


362. // get suffix and create a corresponding serializer
363. if ($type=='rdf') {
364. // Import Package Syntax
365. include_once(RDFAPI_INCLUDE_DIR.PACKAGE_SYNTAX_RDF);
366. $ser=new RdfSerializer();
367. }elseif ($type=='nt') {
368. // Import Package Syntax
369. include_once(RDFAPI_INCLUDE_DIR.PACKAGE_SYNTAX_N3);
370. $ser=new NTripleSerializer();
371. }elseif ($type=='n3') {
372. // Import Package Syntax
373. include_once(RDFAPI_INCLUDE_DIR.PACKAGE_SYNTAX_N3);
374. $ser=new N3Serializer();
375. }else {
376. print ('Serializer type not properly defined. Use the strings "rdf","n3" or "nt".');
377. return false;
378. };

380. return $ser->saveAs($this, $filename);
381. }


384. /**
385. * Tests if the MemModel contains the given triple.
386. * TRUE if the triple belongs to the MemModel;
387. * FALSE otherwise.
388. *
389. * @param    object Statement    &$statement
390. * @return    boolean
391. * @access    public
392. */
393. function contains(&$statement) {

395. // no index ->linear contains
396. if ($this->indexed==-1){
397. foreach($this->triples as $value) {
398. if ($value->equals($statement)){
399. return TRUE; }
400. }
401. return false;
402. }
403. if ($this->indexed==0){
404. $res = $this->_containsIndex($statement,4);
405. return $res;
406. }else{
407. return $this->_containsIndex($statement,$this->indexed);
408. }
409. }


412. /**
413. * Determine if all of the statements in a model are also contained in this MemModel.
414. * True if all of the statements in $model are also contained in this MemModel and false otherwise.
415. *
416. * @param    object Model    &$model
417. * @return    boolean
418. * @access    public
419. */
420. function containsAll(&$model) {

422. if (is_a($model, 'MemModel')) {

424. foreach($model->triples as $statement)
425. if(!$this->contains($statement))
426. return FALSE;
427. return TRUE;

429. }elseif (is_a($model, 'DbModel'))

431. return $model->containsAll($this);

433. $errmsg = RDFAPI_ERROR . '(class: MemModel; method: containsAll): Model expected.';
434. trigger_error($errmsg, E_USER_ERROR);
435. }

437. /**
438. * Determine if any of the statements in a model are also contained in this MemModel.
439. * True if any of the statements in $model are also contained in this MemModel and false otherwise.
440. *
441. * @param    object Model    &$model
442. * @return    boolean
443. * @access    public
444. */
445. function containsAny(&$model) {

447. if (is_a($model, 'MemModel')) {

449. foreach($model->triples as $modelStatement)
450. if($this->contains($modelStatement))
451. return TRUE;
452. return FALSE;

454. }elseif (is_a($model, 'DbModel'))

456. return $model->containsAny($this);

458. $errmsg = RDFAPI_ERROR . '(class: MemModel; method: containsAll): Model expected.';
459. trigger_error($errmsg, E_USER_ERROR);
460. }


463. /**
464. * Builds a search index for the statements in the MemModel.
465. * The index is used by the find(),contains(),add() and remove() functions.
466. * Performance example using a model with 43000 statements on a Linux machine:
467. * Find without index takes 1.7 seconds.
468. * Indexing takes 1.8 seconds.
469. * Find with index takes 0.001 seconds.
470. * So if you want to query a model more then once, build a index first.
471. * The defaultindex is indices over subject, predicate, object seperate.
472. *
473. * mode = 0    : indices over subject,predicate,object (default)
474. * mode = 1     : index over subject+predicate+object
475. * mode = 2      : index over subject+predicate
476. * mode = 3     : index over subject+object
477. *
478. * @param     int $mode
479. * @access    public
480. */
481. function index($mode) {

483. unset($this->indexArr);
484. $this->indexArr=array();
485. switch($mode){
486. // unset indices
487. case -1:
488. $this->indexed=-1;
489. unset($this->indexArr);
490. break;
491. // index over SPO
492. case 0:
493. $this->indexed=0;
494. foreach($this->triples as $k => $t) {
495. // index over S
496. $this->_indexOpr($t,$k,4,1);
497. // index over P
498. $this->_indexOpr($t,$k,5,1);
499. // index over O
500. $this->_indexOpr($t,$k,6,1);
501. }
502. break;
503. default:
504. $this->indexed=$mode;
505. foreach($this->triples as $k => $t) {
506. $this->_indexOpr($t,$k,$this->indexed,1);
507. }
508. break;
509. }
510. }


513. /**
514. * Returns     true if there is an index, false if not.
515. *
516. * @return    boolean
517. * @access    public
518. */
519. function isIndexed() {
520. if($this->indexed!=-1){
521. return TRUE;
522. }else{
523. return FALSE;
524. }
525. }

527. /**
528. * Returns the indextype:
529. * -1 if there is no index, 0 if there are indices over S,P,O(separate),
530. * 1 if there is an index over SPO, 2 if there is an index over SP and 3 if
531. * there is an index over SO.
532. *
533. * @return int
534. * @access public
535. *
536. */
537. function getIndexType(){
538. return $this->indexed;
539. }

541. /**
542. * General method to search for triples.
543. * NULL input for any parameter will match anything.
544. * Example:  $result = $m->find( NULL, NULL, $node );
545. * Finds all triples with $node as object.
546. * Returns an empty MemModel if nothing is found.
547. *
548. * @param    object Node    $subject
549. * @param    object Node    $predicate
550. * @param    object Node    $object
551. * @return    object MemModel
552. * @access    public
553. * @throws    PhpError
554. */

556. function find($subject,$predicate,$object) {

558. if (
559. (!is_a($subject, 'Resource') && $subject != NULL) ||
560. (!is_a($predicate, 'Resource') && $predicate != NULL) ||
561. (!is_a($object, 'Node') && $object != NULL)
562. ) {
563. $errmsg = RDFAPI_ERROR . '(class: MemModel; method: find): Parameters must be subclasses of Node or NULL';
564. trigger_error($errmsg, E_USER_ERROR);
565. }

567. $res = new MemModel($this->getBaseURI());
568. $res->indexed=-1;

570. if($this->isEmpty())
571. return $res;

573. if($subject == NULL && $predicate == NULL && $object == NULL)
574. return $this;

576. switch($this->indexed){
577. case 1:
578. if($subject!=NULL && $predicate != NULL && $object != NULL){
579. $pos=$subject->getLabel().$predicate->getLabel().$object->getLabel();
580. return $this->_findInIndex($pos,$subject,$predicate,$object,1);
581. }else{
582. break;
583. }

585. case 2:
586. if($subject!=NULL && $predicate != NULL){
587. $pos=$subject->getLabel().$predicate->getLabel();
588. return $this->_findInIndex($pos,$subject,$predicate,$object,2);
589. }else{
590. break;
591. }

593. case 3:
594. if($subject!=NULL && $object != NULL){
595. $pos=$subject->getLabel().$object->getLabel();
596. return $this->_findInIndex($pos,$subject,$predicate,$object,3);
597. }else{
598. break;
599. }
600. case 0:
601. if($subject!= null){
602. $pos=$subject->getLabel();
603. return $this->_findInIndex($pos,$subject,$predicate,$object,4);
604. }
605. if($predicate!= null){
606. $pos=$predicate->getLabel();
607. return $this->_findInIndex($pos,$subject,$predicate,$object,5);
608. }
609. if($object!= null){
610. $pos=$object->getLabel();
611. return $this->_findInIndex($pos,$subject,$predicate,$object,6);
612. }
613. }
614. // if no index: linear search
615. foreach($this->triples as $value) {
616. if ($this->matchStatement($value, $subject, $predicate, $object))
617. $res->add($value);
618. }
619. return $res;

621. }


627. /**
628. * Method to search for triples using Perl-style regular expressions.
629. * NULL input for any parameter will match anything.
630. * Example:  $result = $m->find_regex( NULL, NULL, $regex );
631. * Finds all triples where the label of the object node matches the regular expression.
632. * Returns an empty MemModel if nothing is found.
633. *
634. * @param    string    $subject_regex
635. * @param    string    $predicate_regex
636. * @param    string    $object_regex
637. * @return    object MemModel
638. * @access    public
639. */
640. function findRegex($subject_regex, $predicate_regex, $object_regex) {

642. $res = new MemModel($this->getBaseURI());

644. if($this->size() == 0)
645. return $res;

647. if($subject_regex == NULL && $predicate_regex == NULL && $object_regex == NULL)
648. return $this;

650. foreach($this->triples as $value) {
651. if (
652. ($subject_regex == NULL || preg_match($subject_regex, $value->subj->getLabel())) &&
653. ($predicate_regex == NULL || preg_match($predicate_regex, $value->pred->getLabel())) &&
654. ($object_regex == NULL || preg_match($object_regex, $value->obj->getLabel()))
655. ) $res->add($value);
656. }

658. return $res;

660. }

662. /**
663. * Returns all tripels of a certain vocabulary.
664. * $vocabulary is the namespace of the vocabulary inluding a # : / char at the end.
665. * e.g. http://www.w3.org/2000/01/rdf-schema#
666. * Returns an empty MemModel if nothing is found.
667. *
668. * @param    string    $vocabulary
669. * @return    object MemModel
670. * @access    public
671. */
672. function findVocabulary($vocabulary) {

674. if($this->size() == 0)
675. return $res;
676. if($vocabulary == NULL || $vocabulary == '')
677. return $this;

679. $res = new MemModel($this->getBaseURI());
680. if($this->indexed==0){
681. foreach($this->indexArr[5] as $key => $value){
682. $pos=strpos($key,'#')+1;
683. if(substr($key,0,$pos)==$vocabulary){
684. for($i=1;$i<=$value[0];$i++){
685. $res->add($this->triples[$value[$i]]);
686. }
687. }
688. }
689. return $res;
690. }else{
691. // Import Package Utility
692. include_once(RDFAPI_INCLUDE_DIR.PACKAGE_UTILITY);
693. foreach($this->triples as $value) {
694. if (RDFUtil::getNamespace($value->getPredicate()) == $vocabulary)
695. $res->add($value);
696. }
697. return $res;
698. }
699. }

701. /**
702. * Searches for triples and returns the first matching statement.
703. * NULL input for any parameter will match anything.
704. * Example:  $result = $m->findFirstMatchingStatement( NULL, NULL, $node );
705. * Returns the first statement of the MemModel where the object equals $node.
706. * Returns an NULL if nothing is found.
707. * You can define an offset to search for. Default = 0
708. *
709. * @param    object Node    $subject
710. * @param    object Node    $predicate
711. * @param    object Node    $object
712. * @param    integer    $offset
713. * @return    object Statement
714. * @access    public
715. */
716. function findFirstMatchingStatement($subject, $predicate, $object, $offset = 0) {

718. $currentOffset = 0;
719. for($i=0;$i<=$offset;$i++)
720. {
721. $res = $this->findFirstMatchOff($subject, $predicate, $object, $currentOffset);
722. $currentOffset=$res+1;
723. }
724. if ($res != -1) {
725. return $this->triples[$res];
726. } else {
727. return NULL;
728. }
729. }


734. /**
735. * Searches for triples and returns the first matching statement from a given offset.
736. * This method is used by the util/findIterator. NULL input for any parameter will match anything.
737. * Example:  $result = $m->findFirstMatchingStatement( NULL, NULL, $node, $off );
738. * Returns the position of the first statement of the MemModel where the object equals $node from the given
739. * offset.
740. * Returns an -1 if nothing is found.
741. *
742. * @param    object Node    $subject
743. * @param    object Node    $predicate
744. * @param    object Node    $object
745. * @param int         $off
746. * @return    int
747. * @access    private
748. */
749. function findFirstMatchOff($subject,$predicate, $object,$off) {

751. if (
752. (!is_a($subject, 'Resource') && $subject != NULL) ||
753. (!is_a($predicate, 'Resource') && $predicate != NULL) ||
754. (!is_a($object, 'Node') && $object != NULL)
755. ) {
756. $errmsg = RDFAPI_ERROR . '(class: MemModel; method: find): Parameters must be subclasses of Node or NULL';
757. trigger_error($errmsg, E_USER_ERROR);
758. }

760. $match=-1;
761. $ind=$this->indexed;
762. if($subject == NULL && $predicate == NULL && $object == NULL)
763. {
764. foreach ($this->triples as $key => $statement)
765. {
766. if ($key >= $off)
767. return $key;
768. }
769. return -1;
770. }

772. switch($ind){
773. case 1:
774. if($subject!=NULL && $predicate != NULL && $object != NULL){
775. $pos=$subject->getLabel().$predicate->getLabel().$object->getLabel();
776. return $this->_findMatchIndex($pos,$subject,$predicate,$object,1,$off);
777. }else{
778. break;
779. }

781. case 2:
782. if($subject!=NULL && $predicate != NULL){
783. $pos=$subject->getLabel().$predicate->getLabel();
784. return $this->_findMatchIndex($pos,$subject,$predicate,$object,2,$off);
785. }else{
786. break;
787. }

789. case 3:
790. if($subject!=NULL && $object != NULL){
791. $pos=$subject->getLabel().$object->getLabel();
792. return $this->_findMatchIndex($pos,$subject,$predicate,$object,3,$off);
793. }else{
794. break;
795. }
796. case 0:
797. if($subject!= null){
798. $pos=$subject->getLabel();
799. return $this->_findMatchIndex($pos,$subject,$predicate,$object,4,$off);
800. }
801. if($predicate!= null){
802. $pos=$predicate->getLabel();
803. return $this->_findMatchIndex($pos,$subject,$predicate,$object,5,$off);
804. }
805. if($object!= null){
806. $pos=$object->getLabel();
807. return $this->_findMatchIndex($pos,$subject,$predicate,$object,6,$off);
808. }
809. break;
810. }
811. // if no index: linear search
812. foreach($this->triples as $key => $value){
813. if ($this->matchStatement($value, $subject, $predicate, $object)){
814. if($off<=$key){
815. $match=$key;
816. break;
817. }
818. }
819. }
820. return $match;
821. }


824. /**
825. * Searches for triples and returns the number of matches.
826. * NULL input for any parameter will match anything.
827. * Example:  $result = $m->findCount( NULL, NULL, $node );
828. * Finds all triples with $node as object.
829. *
830. * @param    object Node    $subject
831. * @param    object Node    $predicate
832. * @param    object Node    $object
833. * @return    integer
834. * @access    public
835. */
836. function findCount($subject, $predicate, $object) {

838. $res = $this->find($subject, $predicate, $object);
839. return $res->size();

841. }


844. /**
845. * Perform an RDQL query on this MemModel.
846. * This method returns an associative array of variable bindings.
847. * The values of the query variables can either be RAP's objects (instances of Node)
848. * if $returnNodes set to TRUE, or their string serialization.
849. *
850. * @access    public
851. * @param string $queryString
852. * @param boolean $returnNodes
853. * @return  array   [][?VARNAME] = object Node  (if $returnNodes = TRUE)
854. *       OR  array   [][?VARNAME] = string
855. *
856. */
857. function rdqlQuery($queryString, $returnNodes = TRUE) {

859. // Import RDQL Package
860. include_once(RDFAPI_INCLUDE_DIR.PACKAGE_RDQL);

862. $parser = new RdqlParser();
863. $parsedQuery =& $parser->parseQuery($queryString);

865. // this method can only query this MemModel
866. // if another model was specified in the from clause throw an error
867. if (isset($parsedQuery['sources'][1])) {
868. $errmsg = RDFAPI_ERROR . '(class: MemModel; method: rdqlQuery):';
869. $errmsg .= ' this method can only query this MemModel';
870. trigger_error($errmsg, E_USER_ERROR);
871. }

873. $engine = new RdqlMemEngine();
874. $res =& $engine->queryModel($this, $parsedQuery, $returnNodes);

876. return $res;
877. }

879. /**
880. * Perform an RDQL query on this MemModel.
881. * This method returns an RdqlResultIterator of variable bindings.
882. * The values of the query variables can either be RAP's objects (instances of Node)
883. * if $returnNodes set to TRUE, or their string serialization.
884. *
885. * @access    public
886. * @param string $queryString
887. * @param boolean $returnNodes
888. * @return  object RdqlResultIterator = with values as object Node  (if $returnNodes = TRUE)
889. *       OR  object RdqlResultIterator = with values as strings if (if $returnNodes = FALSE)
890. *
891. */
892. function rdqlQueryAsIterator($queryString, $returnNodes = TRUE) {
893. // Import RDQL Package
894. include_once(RDFAPI_INCLUDE_DIR.PACKAGE_RDQL);
895. return new RdqlResultIterator($this->rdqlQuery($queryString, $returnNodes));
896. }

898. /**
899. * General method to replace nodes of a MemModel.
900. * NULL input for any parameter will match nothing.
901. * Example:  $m->replace($node, NULL, $node, $replacement);
902. * Replaces all $node objects beeing subject or object in
903. * any triple of the MemModel with the $needle node.
904. *
905. * @param    object Node    $subject
906. * @param    object Node    $predicate
907. * @param    object Node    $object
908. * @param    object Node    $replacement
909. * @access    public
910. * @throws    PhpError
911. */
912. function replace($subject, $predicate, $object, $replacement) {

914. if (
915. (!is_a($replacement, 'Node')) ||
916. (!is_a($subject, 'Resource') && $subject != NULL) ||
917. (!is_a($predicate, 'Resource') && $predicate != NULL) ||
918. (!is_a($object, 'Node') && $object != NULL)
919. ) {
920. $errmsg = RDFAPI_ERROR . '(class: MemModel; method: replace): Parameters must be subclasses of Node or NULL';
921. trigger_error($errmsg, E_USER_ERROR);
922. }

924. if($this->size() == 0)
925. break;
926. foreach($this->triples as $key => $value) {
927. if ($this->triples[$key]->subj->equals($subject)) {
928. $this->triples[$key]->subj = $replacement;
929. }
930. if ($this->triples[$key]->pred->equals($predicate))
931. $this->triples[$key]->pred = $replacement;
932. if ($this->triples[$key]->obj->equals($object))
933. $this->triples[$key]->obj = $replacement;

935. }
936. $this->index($this->indexed);
937. }


940. /**
941. * Internal method that checks, if a statement matches a S, P, O or NULL combination.
942. * NULL input for any parameter will match anything.
943. *
944. * @param    object Statement    $statement
945. * @param    object Node    $subject
946. * @param    object Node    $predicate
947. * @param    object Node    $object
948. * @return    boolean
949. * @access    private
950. */
951. function matchStatement($statement, $subject, $predicate, $object)  {

953. if(($subject != NULL) AND !($statement->subj->equals($subject)))
954. return false;

956. if($predicate != NULL && !($statement->pred->equals($predicate)))
957. return false;

959. if($object != NULL && !($statement->obj->equals($object)))
960. return false;

962. return true;
963. }


968. /**
969. * Checks if two models are equal.
970. * Two models are equal if and only if the two RDF graphs they represent are isomorphic.
971. *
972. * Warning: This method doesn't work correct with models where the same blank node has different
973. * identifiers in the two models. We will correct this in a future version.
974. *
975. * @access    public
976. * @param        object    model &$that
977. * @throws    phpErrpr
978. * @return    boolean
979. */

981. function equals(&$that)  {

983. if (!is_a($that, 'Model')) {
984. $errmsg = RDFAPI_ERROR . '(class: MemModel; method: equals): Model expected.';
985. trigger_error($errmsg, E_USER_ERROR);
986. }

988. if ($this->size() != $that->size())
989. return FALSE;

991. if (!$this->containsAll($that))
992. return FALSE;
993. return TRUE;
994. }

996. /**
997. * Returns a new MemModel that is the set-union of the MemModel with another model.
998. * Duplicate statements are removed. If you want to allow duplicates, use addModel() which is much faster.
999. *
1000. * The result of taking the set-union of two or more RDF graphs (i.e. sets of triples)
1001. * is another graph, which we will call the merge of the graphs.
1002. * Each of the original graphs is a subgraph of the merged graph. Notice that when forming
1003. * a merged graph, two occurrences of a given uriref or literal as nodes in two different
1004. * graphs become a single node in the union graph (since by definition they are the same
1005. * uriref or literal) but blank nodes are not 'merged' in this way; and arcs are of course
1006. * never merged. In particular, this means that every blank node in a merged graph can be
1007. * identified as coming from one particular graph in the original set of graphs.
1008. *
1009. * Notice that one does not, in general, obtain the merge of a set of graphs by concatenating
1010. * their corresponding N-triples documents and constructing the graph described by the merged
1011. * document, since if some of the documents use the same node identifiers, the merged document
1012. * will describe a graph in which some of the blank nodes have been 'accidentally' merged.
1013. * To merge Ntriples documents it is necessary to check if the same nodeID is used in two or
1014. * more documents, and to replace it with a distinct nodeID in each of them, before merging the
1015. * documents. (Not implemented yet !!!!!!!!!!!)
1016. *
1017. * @param    object Model    $model
1018. * @return    object MemModel
1019. * @access    public
1020. * @throws phpErrpr
1021. *
1022. */
1023. function & unite(&$model)  {

1025. if (!is_a($model, 'Model')) {
1026. $errmsg = RDFAPI_ERROR . '(class: MemModel; method: unite): Model expected.';
1027. trigger_error($errmsg, E_USER_ERROR);
1028. }

1030. $res = $this;

1032. if (is_a($model, 'MemModel')) {
1033. include_once(RDFAPI_INCLUDE_DIR.PACKAGE_UTILITY);
1034. $stateIt=new StatementIterator($model);
1035. while($statement=$stateIt->next())
1036. {
1037. $res->addWithoutDuplicates($statement);
1038. }
1039. }

1041. elseif (is_a($model, 'DbModel')) {
1042. $memModel =& $model->getMemModel();
1043. foreach($memModel->triples as $value)
1044. $res->addWithoutDuplicates($value);
1045. }

1047. return $res;
1048. }

1050. /**
1051. * Returns a new MemModel that is the subtraction of another model from this MemModel.
1052. *
1053. * @param    object Model    $model
1054. * @return    object MemModel
1055. * @access    public
1056. * @throws phpErrpr
1057. */

1059. function & subtract(&$model)  {

1061. if (!is_a($model, 'Model')) {
1062. $errmsg = RDFAPI_ERROR . '(class: MemModel; method: subtract): Model expected.';
1063. trigger_error($errmsg, E_USER_ERROR);
1064. }

1066. $res = $this;


1069. if (is_a($model, 'MemModel'))
1070. {
1071. include_once(RDFAPI_INCLUDE_DIR.PACKAGE_UTILITY);
1072. $stateIt=new StatementIterator($model);
1073. while($statement=$stateIt->next())
1074. {
1075. $res->remove($statement);
1076. }
1077. }
1078. elseif (is_a($model, 'DbModel'))
1079. {
1080. $memModel =& $model->getMemModel();
1081. foreach($memModel->triples as $value)
1082. $res->remove($value);
1083. }


1086. return $res;
1087. }

1089. /**
1090. * Returns a new MemModel containing all the statements which are in both this MemModel and another.
1091. *
1092. * @param    object Model    $model
1093. * @return    object MemModel
1094. * @access    public
1095. * @throws phpErrpr
1096. */
1097. function & intersect(&$model)  {

1099. if (!is_a($model, 'Model')) {
1100. $errmsg = RDFAPI_ERROR . '(class: MemModel; method: intersect: Model expected.';
1101. trigger_error($errmsg, E_USER_ERROR);
1102. }

1104. $res = new MemModel($this->getBaseURI());

1106. if (is_a($model, 'DbModel') || is_a($model, 'RDFSBModel'))
1107. {
1108. $memModel =& $model->getMemModel();
1109. foreach($memModel->triples as $value) {
1110. if ($this->contains($value))
1111. $res->add($value);
1112. }
1113. }

1115. elseif (is_a($model, 'MemModel'))
1116. {
1117. foreach($model->triples as $value) {
1118. if ($this->contains($value))
1119. $res->add($value);
1120. }
1121. }


1125. return $res;
1126. }


1129. /**
1130. * Adds another model to this MemModel.
1131. * Duplicate statements are not removed.
1132. * If you don't want duplicates, use unite().
1133. * If any statement of the model to be added to this model contains a blankNode
1134. * with an identifier already existing in this model, a new blankNode is generated.
1135. *
1136. * @param    object Model    $model
1137. * @access    public
1138. * @throws phpErrpr
1139. *
1140. */
1141. function addModel(&$model)  {

1143. if (!is_a($model, 'Model')) {
1144. $errmsg = RDFAPI_ERROR . '(class: MemModel; method: addModel): Model expected.';
1145. trigger_error($errmsg, E_USER_ERROR);
1146. }

1148. $blankNodes_tmp = array();

1150. if (is_a($model, 'MemModel')) {
1151. include_once(RDFAPI_INCLUDE_DIR.PACKAGE_UTILITY);
1152. $stateIt=new StatementIterator($model);
1153. while($statement=$stateIt->next())
1154. {
1155. $this->_addStatementFromAnotherModel($statement, $blankNodes_tmp);
1156. };
1157. $this->addParsedNamespaces($model->getParsedNamespaces());
1158. }

1160. elseif (is_a($model, 'DbModel')) {
1161. $memModel =& $model->getMemModel();
1162. foreach($memModel->triples as $value)
1163. $this->_addStatementFromAnotherModel($value, $blankNodes_tmp);
1164. }
1165. $this->index($this->indexed);
1166. }


1169. /**
1170. * Reifies the MemModel.
1171. * Returns a new MemModel that contains the reifications of all statements of this MemModel.
1172. *
1173. * @access    public
1174. * @return    object    MemModel
1175. */
1176. function & reify() {
1177. $res = new MemModel($this->getBaseURI());

1179. $stateIt=$this->getStatementIterator();
1180. while($statement=$stateIt->next())
1181. {
1182. $pointer =& $statement->reify($res);
1183. $res->addModel($pointer);
1184. };

1186. return $res;
1187. }

1189. /**
1190. * Returns a StatementIterator for traversing the MemModel.
1191. * @access    public
1192. * @return    object    StatementIterator
1193. */
1194. function & getStatementIterator() {
1195. // Import Package Utility
1196. include_once(RDFAPI_INCLUDE_DIR.PACKAGE_UTILITY);

1198. return new StatementIterator($this);
1199. }

1201. /**
1202. * Returns a FindIterator for traversing the MemModel.
1203. * @access    public
1204. * @return    object    FindIterator
1205. */
1206. function & findAsIterator($sub=null,$pred=null,$obj=null) {
1207. // Import Package Utility
1208. include_once(RDFAPI_INCLUDE_DIR.PACKAGE_UTILITY);

1210. return new FindIterator($this,$sub,$pred,$obj);
1211. }

1213. /**
1214. * Returns a FindIterator for traversing the MemModel.
1215. * @access    public
1216. * @return    object    FindIterator
1217. */
1218. function & iterFind($sub=null,$pred=null,$obj=null) {
1219. // Import Package Utility
1220. include_once(RDFAPI_INCLUDE_DIR.PACKAGE_UTILITY);

1222. return new IterFind($this,$sub,$pred,$obj);
1223. }


1226. /**
1227. * Returns the models namespaces.
1228. *
1229. * @author   Tobias Gau� <tobias.gauss@web.de>
1230. * @access   public
1231. * @return   Array
1232. */
1233. function getParsedNamespaces(){
1234. if(count($this->parsedNamespaces)!=0){
1235. return $this->parsedNamespaces;
1236. }else{
1237. return false;
1238. }
1239. }


1243. /**
1244. * Adds the namespaces to the model. This method is called by
1245. * the parser. !!!! addParsedNamespaces() not overwrites manual
1246. * added namespaces in the model !!!!
1247. *
1248. * @author   Tobias Gau� <tobias.gauss@web.de>
1249. * @access   public
1250. * @param    Array $newNs
1251. */
1252. function addParsedNamespaces($newNs){
1253. if($newNs)
1254. $this->parsedNamespaces = $this->parsedNamespaces + $newNs;
1255. }


1258. /**
1259. * Adds a namespace and prefix to the model.
1260. *
1261. * @author   Tobias Gau� <tobias.gauss@web.de>
1262. * @access   public
1263. * @param    String
1264. * @param    String
1265. */
1266. function addNamespace($prefix, $nmsp){
1267. $this->parsedNamespaces[$nmsp]=$prefix;
1268. }

1270. /**
1271. * removes a single namespace from the model
1272. *
1273. * @author   Tobias Gau� <tobias.gauss@web.de>
1274. * @access   public
1275. * @param    String $nmsp
1276. */
1277. function removeNamespace($nmsp){
1278. if(isset($this->parsedNamespaces[$nmsp])){
1279. unset($this->parsedNamespaces[$nmsp]);
1280. return true;
1281. }else{
1282. return false;
1283. }
1284. }


1288. /**
1289. * Close the MemModel and free up resources held.
1290. *
1291. * @access    public
1292. */
1293. function close() {
1294. unset( $baseURI );
1295. unset( $triples );
1296. }

1298. // =============================================================================
1299. // *************************** helper functions ********************************
1300. // =============================================================================

1302. /**
1303. * Checks if $statement is in index
1304. *
1305. * @param  int $ind
1306. * @param  Statement &$statement
1307. * @return boolean
1308. * @access private
1309. */
1310. function _containsIndex(&$statement,$ind){
1311. switch($ind){
1312. case 4:
1313. $sub=$statement->getSubject();
1314. $pos=$sub->getLabel();
1315. break;
1316. case 1:
1317. $sub=$statement->getSubject();
1318. $pred=$statement->getPredicate();
1319. $obj=$statement->getObject();
1320. $pos=$sub->getLabel().$pred->getLabel().$obj->getLabel();
1321. break;
1322. case 2:
1323. $sub=$statement->getSubject();
1324. $pred=$statement->getPredicate();
1325. $pos=$sub->getLabel().$pred->getLabel();
1326. break;
1327. case 3:
1328. $sub=$statement->getSubject();
1329. $obj=$statement->getObject();
1330. $pos=$sub->getLabel().$obj->getLabel();
1331. break;
1332. }

1334. if (!isset($this->indexArr[$ind][$pos]))
1335. return FALSE;
1336. foreach ($this->indexArr[$ind][$pos] as $key => $value) {
1337. $t=$this->triples[$value];
1338. if ($t->equals($statement))
1339. return TRUE;
1340. }
1341. return FALSE;
1342. }


1348. /**
1349. * finds a statement in an index. $pos is the Position in the index
1350. * and $ind the adequate searchindex
1351. *
1352. * @param    String            $pos
1353. * @param    Object Subject    &$subject
1354. * @param    Object Predicate  &$predicate
1355. * @param    Object Object     &$object
1356. * @param    int                 &ind
1357. * @return   MemModel          $res
1358. * @access   private
1359. */
1360. function _findInIndex($pos,&$subject,&$predicate,&$object,$ind){
1361. $res = new MemModel($this->getBaseURI());
1362. $res->indexed=-1;
1363. if (!isset($this->indexArr[$ind][$pos]))
1364. return $res;
1365. foreach($this->indexArr[$ind][$pos] as $key =>$value){
1366. $t=$this->triples[$value];
1367. if ($this->matchStatement($t,$subject,$predicate,$object))
1368. $res->add($t);
1369. }
1370. return $res;
1371. }
1372. /**
1373. * adds/removes a statement into/from an index.
1374. * mode=0 removes the statement from the index;
1375. * mode=1 adds the statement into the index.
1376. * returns the statements position.
1377. *
1378. * @param Object Statement &$statement
1379. *    @param int              $k
1380. *    @param int              $ind
1381. * @param int                $mode
1382. * @return int             $k
1383. * @access private
1384. */
1385. function _indexOpr(&$statement,$k,$ind,$mode){
1386. // determine position in adequate index
1387. switch($ind){
1388. case 1:
1389. $s=$statement->getSubject();
1390. $p=$statement->getPredicate();
1391. $o=$statement->getObject();
1392. $pos=$s->getLabel().$p->getLabel().$o->getLabel();
1393. break;
1394. case 2:
1395. $s=$statement->getSubject();
1396. $p=$statement->getPredicate();
1397. $pos=$s->getLabel().$p->getLabel();
1398. break;
1399. case 3:
1400. $s=$statement->getSubject();
1401. $o=$statement->getObject();
1402. $pos=$s->getLabel().$o->getLabel();
1403. break;
1404. case 4:
1405. $s=$statement->getSubject();
1406. $pos=$s->getLabel();
1407. break;
1408. case 5:
1409. $p=$statement->getPredicate();
1410. $pos=$p->getLabel();
1411. break;
1412. case 6:
1413. $o=$statement->getObject();
1414. $pos=$o->getLabel();
1415. break;
1416. }
1417. switch($mode){
1418. // add in Index
1419. case 1:
1420. if(isset($this->indexArr[$ind][$pos])){
1421. $this->indexArr[$ind][$pos][] = $k;
1422. }else{
1423. $this->indexArr[$ind][$pos][0] = $k;
1424. }
1425. break;
1426. // remove from Index
1427. case 0:
1428. $subject=$statement->getSubject();
1429. $predicate=$statement->getPredicate();
1430. $object=$statement->getObject();
1431. $k=-1;
1432. if(!isset($this->indexArr[$ind][$pos])){
1433. return -1;
1434. }
1435. $num=count($this->indexArr[$ind][$pos]);
1436. foreach($this->indexArr[$ind][$pos] as $key => $value){
1437. $t=$this->triples[$value];
1438. if($this->matchStatement($t,$subject,$predicate,$object)){
1439. $k=$value;
1440. if($num==1){
1441. unset($this->indexArr[$ind][$pos]);
1442. }else{
1443. unset($this->indexArr[$ind][$pos][$key]);
1444. }
1445. return $k;
1446. }
1447. }
1448. break;
1449. }
1450. return $k;
1451. }


1454. /**
1455. * finds next or previous matching statement.
1456. * Returns Position in model or -1 if there is no match.
1457. *
1458. *
1459. * @param        String
1460. * @param     object  Subject
1461. * @param        object    Predicate
1462. * @param     object  Object
1463. * @param     integer
1464. * @param     integer
1465. * @return    integer
1466. * @access    private
1467. */
1468. function _findMatchIndex($pos,&$s,&$p,&$o,$ind,$off){
1469. $match=-1;
1470. if (!isset($this->indexArr[$ind][$pos])) {
1471. return $match;}
1472. foreach($this->indexArr[$ind][$pos] as $key =>$value){
1473. $t=$this->triples[$value];
1474. if ($this->matchStatement($t,$s,$p,$o)){
1475. if($off <= $value){
1476. $match= $value;
1477. return $match;
1478. }
1479. }
1480. }

1482. return $match;

1484. }


1489. } // end: MemModel


1492. ?>
```

Documentation generated on Thu, 7 Jul 2005 13:42:00 +0200 by phpDocumentor 1.3.0RC3
